# Successes and Lessons Learned From Age-Friendly Community Collaborations: Baystate Health GWEP

**DOI:** 10.1093/geroni/igab046.1907

**Published:** 2021-12-17

**Authors:** Maura Brennan, Rebecca Dobert

**Affiliations:** Baystate Health, Springfield, Massachusetts, United States

## Abstract

Baystate is the largest health system in Western Massachusetts with 4 hospitals, 3 Community Health Centers (CHCs) and a large primary care network. Baystate Medical Center (BMC) is in Springfield, Massachusetts. BMC and the CHCs were the first health care sites nationally to be recognized by the Institute for Healthcare Improvement as “Committed to Care Excellence” in the age friendly movement. Collaboration with a city-wide coalition of community-based organizations led to simultaneous recognition of Baystate as “age friendly” and recognition of the city as both dementia and age friendly. The 3 awards were presented at a Springfield senior center with media coverage and the participation of the mayor and other political leaders. This collaboration persists and the GWEP and coalition partners continue to participate in multiple joint educational and community outreach projects. As a result, the city coalition has added health care to its initial focus on housing and transportation.

